# Endovascular coiling versus neurosurgical clipping in the management of aneurysmal subarachnoid haemorrhage in the elderly: a multicenter cohort study

**DOI:** 10.1007/s10143-024-02325-z

**Published:** 2024-03-01

**Authors:** Keng Siang Lee, Isabel Siow, Lily WY Yang, Aaron SC Foo, John JY Zhang, Ian Mathews, Chun Peng Goh, Colin Teo, Bolem Nagarjun, Vanessa Chen, Sein Lwin, Kejia Teo, Shiong Wen Low, Ira SY Sun, Boon Chuan Pang, Eugene WR Yang, Cunli Yang, Anil Gopinathan, Tseng Tsai Yeo, Vincent DW Nga

**Affiliations:** 1 Division of Neurosurgery, Department of Surgery, National University Health System, Singapore, Singapore; 2 Department of Neurosurgery, King’s College Hospital, London, UK; 3 Department of Basic and Clinical Neurosciences, Wohl Clinical Neuroscience Institute, Institute of Psychiatry, Psychology and Neuroscience (IoPPN), King’s College London, London, UK; 4 Department of Neurology, National Neuroscience Institute (Singapore General Hospital Campus), Singapore, Singapore; 5 Ministry of Health Holdings, Singapore, Singapore; 6 Emergency Medicine Department, National University Hospital, Singapore, Singapore; 7 Division of Neurosurgery, Ng Teng Fong General Hospital, National University Health System, Singapore, Singapore; 8 Department of Neurosurgery, Khoo Teck Puat Hospital, Alexandra Health Private Limited, National University Health System, Singapore, Singapore; 9 Division of Interventional Radiology, Department of Diagnostic Imaging, National University Health System, Singapore, Singapore

**Keywords:** Aneurysm, Subarachnoid hemorrhage, Elderly, Geriatric, Endovascular, Clipping, Cohort study

## Abstract

The comparability of endovascular coiling over neurosurgical clipping has not been firmly established in elderly patients with aneurysmal subarachnoid haemorrhage (aSAH). Data were obtained from all patients with aSAH aged ≥60 across three tertiary hospitals in Singapore from 2014 to 2019. Outcome measures included modified Rankin Scale (mRS) score at 3 and at 6 months, and in-hospital mortality. Of the 134 patients analyzed, 84 (62.7%) underwent coiling and 50 (37.3%) underwent clipping. The endovascular group showed a higher incidence of good mRS score 0–2 at 3 months (OR = 2.45 [95%CI:1.16–5.20];*p* = 0.018), and a lower incidence of in-hospital mortality (OR = 0.31 [95%CI:0.10–0.91];*p* = 0.026). There were no significant difference between the two treatment groups in terms of good mRS score at 6 months (OR = 1.98 [95%CI:0.97–4.04];*p* = 0.060). There were no significant differences in the incidence of complications, such as aneurysm rebleed, delayed hydrocephalus, delayed ischemic neurological deficit and venous thromboembolism between the two treatment groups. However, fewer patients in the coiling group developed large infarcts requiring decompressive craniectomy (OR = 0.32 [95%CI:0.12–0.90];*p* = 0.025). Age, admission WFNS score I–III, and coiling were independent predictors of good functional outcomes at 3 months. Only age and admission WFNS score I–III remained significant predictors of good functional outcomes at 6 months. Endovascular coiling, compared with neurosurgical clipping, is associated with significantly better short term outcomes in carefully selected elderly patients with aSAH. Maximal intervention is recommended for aSAH in the young elderly age group and those with favorable WFNS scores.

## Introduction

Longer life expectancies are leading to aging populations and rising incidence of aneurysmal subarachnoid haemorrhage (aSAH) [1–3]. The advent of minimally invasive aneurysm securing techniques has encouraged a gradual shift in treatment paradigm for this age group to encompass early treatment comprising microsurgical clipping, endovascular coiling and neurointensive care [4, 5]. As a result, clinical practice has also changed and more elderly aSAH patients are being referred to neurosurgical centers [6, 7].

Treatment of elderly patients with aSAH however still remains a clinical challenge [3, 5, 8, 9], largely owing to their higher rate of poor clinical grade on admission, severe aSAH on initial computed tomography (CT) scan, and general complications [10, 11]. Older age within this cohort has also been associated with poor outcomes [5]. The literature reporting outcomes for elderly patients with aSAH is scarce and optimal management of this condition in the elderly remains unclear [4, 9, 10, 12–15].

Whilst the recommendation for endovascular coiling over surgical clipping for SAH in general is relatively well established [16–19], the International Subarachnoid Aneurysm Trial (ISAT) has been the only large, multicenter, RCT that compared neurosurgical clipping with detachable platinum coils in patients with ruptured intracranial aneurysms, who were considered to be suitable for either treatment [16, 17]. However, results of ISAT have continued to generate some criticism, mainly because of its selection bias.

For example, the vast majority of the enrolled patients, had a favourable grade at the time of enrolment, 95% of the aneurysms were in the anterior cerebral circulation, and 90% were smaller than 10 mm. This makes it difficult for clinicians to generalise the results to their own practice. Hence whether the superiority of coiling still prevails in the elderly subgroup remains unconfirmed. Therefore, our study aims to explore the outcomes of endovascular coiling and neurosurgical clipping in a unique cohort of elderly patients with aSAH.

## Methods

### Data collection

This cohort study adhered to the Strengthening the Reporting of Observational Studies in Epidemiology (STROBE) guidelines. Ethics approval was obtained from the institutional review board before commencement of the study. Data were obtained retrospectively from the National University Health System (NUHS) cluster in Singapore which included the National University Hospital, Khoo Teck Puat Hospital and Ng Teng Fong General Hospital, over a period of six years from 2014 to 2019. Electronic medical records were reviewed for all for elderly patients (aged 60 years and above) who had undergone treatment for aSAH, within 24 hours, at our institutions. The definition of ‘elderly’ may vary according to chronological, biological, psychological, and social aspects. In this study patients aged 60 years and above were categorized into the elderly age group in line with the definition established by the World Health Organisation (WHO) and United Nations (UN) [12, 20]. Diagnosis of aSAH was confirmed with a computed tomography head scan or a lumbar puncture. Evidence of aSAH was verified by a neuroradiologist on computed tomography angiography or digital subtraction angiography. Patients with no aSAH, or those who did not undergo treatment were excluded from the analysis.

The collected patient variables included: age, gender, smoking history, and comorbidities (hypertension, hyperlipidaemia, diabetes mellitus, and ischaemic heart disease), and World Federation of Neurosurgical Societies (WFNS) score on admission, dichotomized as good (Grades I – III) and poor (Grades IV – V). The following information about aSAH features was extracted: number of aneurysms, size of aneurysms, and location of subarachnoid haemorrhages. Additionally, information on the treatment modality (coiling or clipping) was obtained. The following information about patient complications was also extracted: aneurysm re-rupture, delayed hydrocephalus, delayed ischaemic neurological deficit, large infarct requiring decompressive craniectomy, and venous thromboembolism.

### Outcome measures

The primary outcome measure was functional outcome defined by the modified Rankin Scale (mRS) at 3 months and 6 months after aSAH. Furthermore, the outcome was dichotomized as favorable (mRS score of 0–2) or unfavorable (mRS score of 3–6). The secondary outcome was in-hospital mortality.

### Statistical analysis

Numerical variables were described as median (interquartile range [IQR]) for non-normal distributions. Normality of data was tested for using the Shapiro-Wilk test. Age was either analyzed as a categorical or a continuous variable. Patients were separated into three age groups: 60–69, 70–79, and ≥80 years. If age was analyzed as a categorical variable, odd ratios (ORs) were compared with the reference category 60–69 years (OR = 1.0) [12]. Comparisons of non-normal numerical data were conducted using the Mann-Whitney U test and comparisons of categorical variables were performed using the Pearson chi-squared test. Multivariable logistic regression was used to identify independent predictors for categorical outcome measures. Covariates were pre-specified based on literature review and expert opinion and included age, admission WFNS score, acute hydrocephalus, aneurysm size and treatment modality.

Additional sensitivity analyses were performed to confirm the robustness of the primary analysis using complete case analysis. Additional series of outcome analyses were performed using multiple imputations by chained equations to address missing covariate and outcome data [21–23]. Multiple imputations were chosen as it can account for uncertainty owing to missing data whilst preserving important data relationships. Ten imputations were used for the relatively modest proportion of missing data [21–23].

Data were collated in Microsoft Excel (Microsoft, Redmond, WA, USA). All statistical analyses were performed using R software version 4.2.1 (R Foundation for Statistical Computing, 2022). P-values less than 0.05 were considered statistically significant.

## Results

### Baseline characteristics

**Fig. 1:**
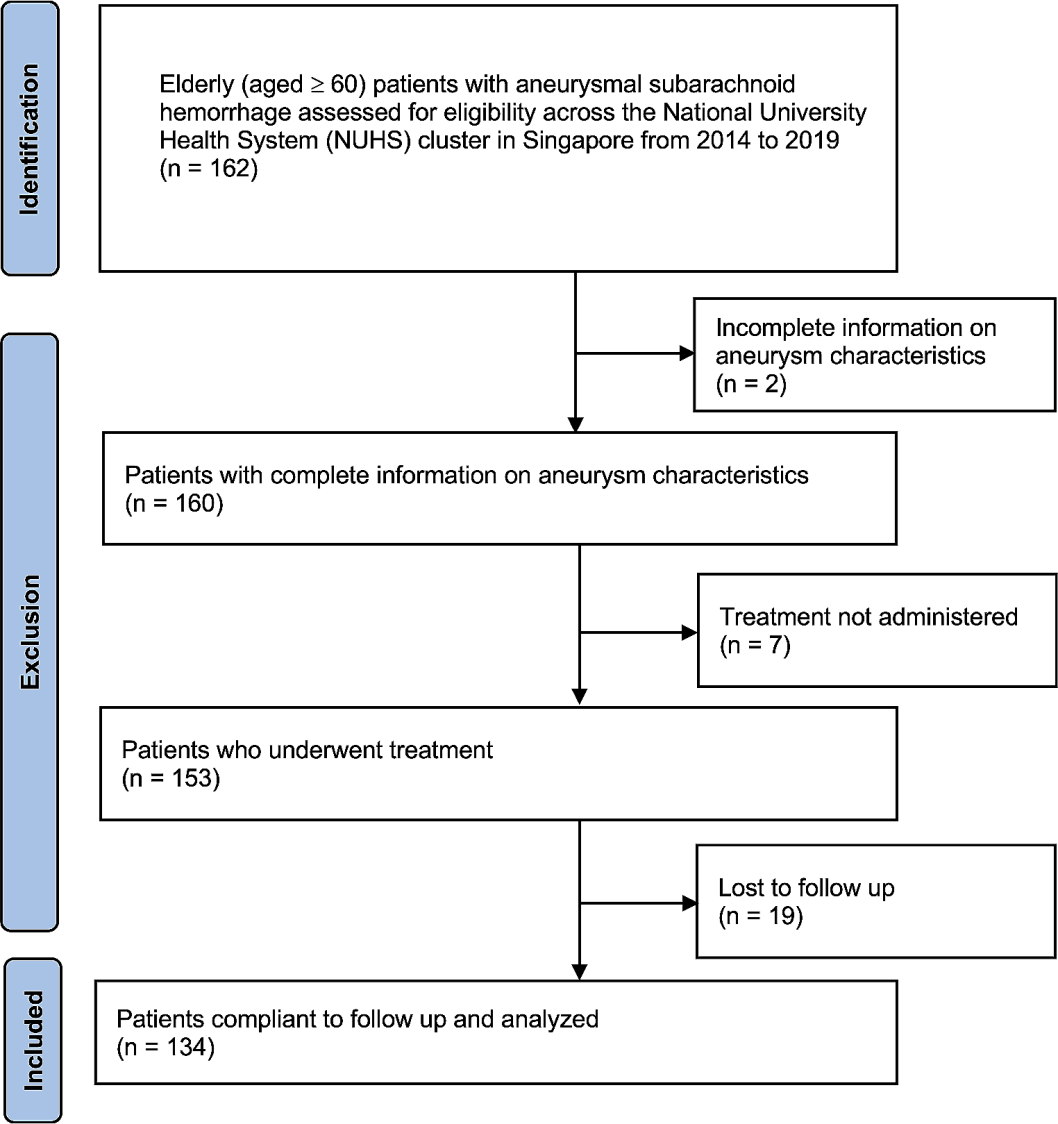
Strengthening the Reporting of Observational Studies in Epidemiology (STROBE) flow chart demonstrating inclusion/exclusion of patients identified across the National University Health System (NUHS) cluster in Singapore from 2014 to 2019

A total of 134 patients were included in our analysis (Fig. 1). The median age of these patients was 68.5 years (IQR 64–74). There were 104 females (77.6%) and 30 males (22.4%), with only six patients (4.5%) having a history of smoking. Eighty-seven patients (64.9%) exhibited at least one comorbidity. The most common comorbidities were hypertension in 75 patients (56.0%), hyperlipidaemia in 58 (43.3%), diabetes mellitus in 18 (13.4%) and ischaemic heart disease in 17 patients (12.7%). On admission, 70 patients (52.2%) had good admission WFNS grade (I – III).

Eighty-four patients (62.7%) underwent coiling whilst 50 patients (37.3%) underwent clipping. Twenty seven (20.1%) patients had multiple aneurysms, 24 (17.9%) with two aneurysms and three (2.2%) with three aneurysms. Ninety one patients (67.9%) had aneurysm < 7 mm in size, with 103 (76.9%) located anteriorly. Table 1 compares the baseline patient and aneurysm characteristics, between the coiling and clipping groups which demonstrates no significant difference.

**Table 1:** Baseline characteristics of the study population

	Endovascular coiling (n = 84)	Neurosurgical clipping (n = 50)	P-value
Age (years)	68 (64–74)	69 (65–74)	0.770
Gender			
Male	21 (25.0)	9 (18.0)	0.347
Female	63 (75.0)	41 (82.0)	
Smoking	4 (4.8)	2 (4.0)	0.837
Comorbidities			
Hypertension	44 (52.4)	31 (62.0)	0.278
Hyperlipidaemia	32 (38.1)	26 (52.0)	0.116
Diabetes Mellitus	10 (11.9)	8 (16.0)	0.501
Ischemic heart disease	12 (14.3)	5 (10.0)	0.471
Acute hydrocephalus requiring EVD	59 (70.2)	42 (84.0)	0.074
Admission WFNS			
I–III	42 (50.0)	28 (56.0)	0.501
IV–V	42 (50.0)	22 (44.0)	
Aneurysm number			
Single	69 (82.1)	38 (76.0)	0.391
Multiple	15 (17.9)	12 (24.0)	
Aneurysm location			
Anterior circulation	60 (71.4)	43 (86.0)	0.053
Posterior circulation	24 (28.6)	7 (14.0)	
Aneurysm size			
< 7mm	61 (72.6)	30 (60.0)	0.130
≥ 7mm	23 (27.4)	20 (40.0)	

All categorical data presented are as n (%) and all numerical data are presented as median (interquartile range). EVD = External Ventricular Drain; WFNS = World Federation of Neurosurgical Societies

### Comparison between endovascular coiling versus clipping

Fifty-five (41.0%) patients had a good functional outcome (mRS score 0–2) at 3 months follow-up, whilst 65 (48.5%) patients had good functional outcome at 6 months follow-up. There were 16 in-hospital mortalities (11.9%) in our cohort of elderly patients. The outcomes and complications sustained between the coiling and clipping groups are presented in Table 2.

**Table 2:** Comparison of outcomes between endovascular and neurosurgical treatment

Outcome	Endovascular coiling (*n* = 84)	Neurosurgical clipping (*n* = 50)	OR (95% CI)	p-value
mRS 0–2 at 3 months	41 (48.8)	14 (28.0)	2.45 (1.16–5.20)	0.018
mRS 0–2 at 6 months	46 (54.8)	19 (38.0)	1.98 (0.97–4.04)	0.060
Aneurysm rebleed	4 (4.8)	3 (6.0)	0.78 (0.17–3.65)	0.755
Delayed hydrocephalus requiring ventriculoperitoneal shunt	23 (27.4)	20 (40.0)	0.57 (0.27–1.19)	0.130
Delayed ischemic neurological deficit	14 (16.7)	4 (8.0)	2.30 (0.71–7.41)	0.155
Large infarct requiring decompressive craniectomy	7 (8.3)	11 (22.0)	0.32 (0.12–0.90)	0.025
Venous thromboembolism	4 (4.8)	1 (2.0)	2.45 (0.27–22.73)	0.415
In-hospital mortality	6 (7.1)	10 (20.0)	0.31 (0.10–0.91)	0.026

All categorical data presented as n (%). mRS = modified Rankin Scale

Neurosurgical clipping group is used as the reference group

The endovascular coiling group showed a higher incidence of good mRS score at 3 months (48.8% in coiling group vs. 28.8% in clipping group; OR = 2.45 [95% CI: 1.16–5.20]; *p* = 0.018), and a lower incidence of in-hospital mortality (7.1% in coiling group vs. 20.0% in clipping group; OR = 0.31 [95%CI: 0.10–0.91]; *p* = 0.026). There were no significant difference between the two treatment groups in terms of good mRS score at 6 months (54.8% in coiling group vs. 38.0% in clipping group; OR = 1.98 [95% CI: 0.97–4.04]; *p* = 0.060). There were no significant differences in the incidence of complications, such as aneurysm rebleed, delayed hydrocephalus, delayed ischemic neurological deficit and venous thromboembolism between the two treatment groups. However, fewer patients in the coiling group developed large infarcts requiring decompressive craniectomy (8.3% in coiling group vs. 22.0% in clipping group; OR = 0.32 [95% CI: 0.12–0.90]; *p* = 0.025).

### Predictors of functional outcomes and mortality

Independent predictors of functional outcome and mortality and their effect sizes are presented in Table 3.

**Table 3:** Multivariable logistic regression for predictors of functional outcome and mortality

Outcome	Predictor	Univariable analysis		Multivariable analysis	
		OR (95% CI)	p-value	OR (95% CI)	p-value
mRS score 0–2 at 3 months	Age*	0.42 (0.24–0.743)	0.003	0.32 (0.17–0.61)	**< 0.001**
	Admission WFNS score I–III^†^	3.28 (1.59–6.79)	0.001	3.81 (1.47–9.88)	**0.009**
	Acute hydrocephalus requiring EVD	0.24 (0.11–0.56)	< 0.001	0.50 (0.18–1.43)	0.197
	Endovascular coiling^‡^	2.45 (1.15–5.20)	0.018	3.31 (1.36–8.02)	**0.012**
mRS score 0–2 at 6 months	Age*	0.52 (0.31–0.87)	0.013	0.46 (0.26–0.79)	**0.005**
	Admission WFNS score I – III^†^	3.47 (1.70–7.06)	< 0.001	3.31 (1.42–7.67.0)	**0.005**
	Acute hydrocephalus requiring EVD	0.31 (0.13–0.72)	0.005	1.64 (0.66–4.72)	0.253
Mortality in hospital	Admission WFNS score I – III^†^	0.18 (0.05–0.65)	0.004	0.15 (0.04–0.62)	**0.008**
	Aneurysm size ≥7 mm^§^	3.17 (1.10–9.17)	0.043	0.69 (0.19–2.5`)	0.577
	Endovascular coiling^‡^	0.31 (0.10–0.91)	0.026	0.28 (0.08–0.95)	**0.041**
	Large infarct requiring decompressive craniectomy	3.68 (1.10–12.20)	0.026	4.23 (0.87–20.833)	0.075
	Delayed hydrocephalus requiring ventriculoperitoneal shunt	0.12 (0.02–0.95)	0.018	0.07 (0.01–0.63)	**0.018**

EVD = External Ventricular Drain; WFNS = World Federation of Neurosurgical Societies

*Age category 60–69 years is used as the reference group

^†^WFNS Grades IV – V is used as the reference group

^‡^Neurosurgical clipping group is used as the reference group

^§^Aneurysm size < 7 mm is used as the reference group

**Fig. 2:**
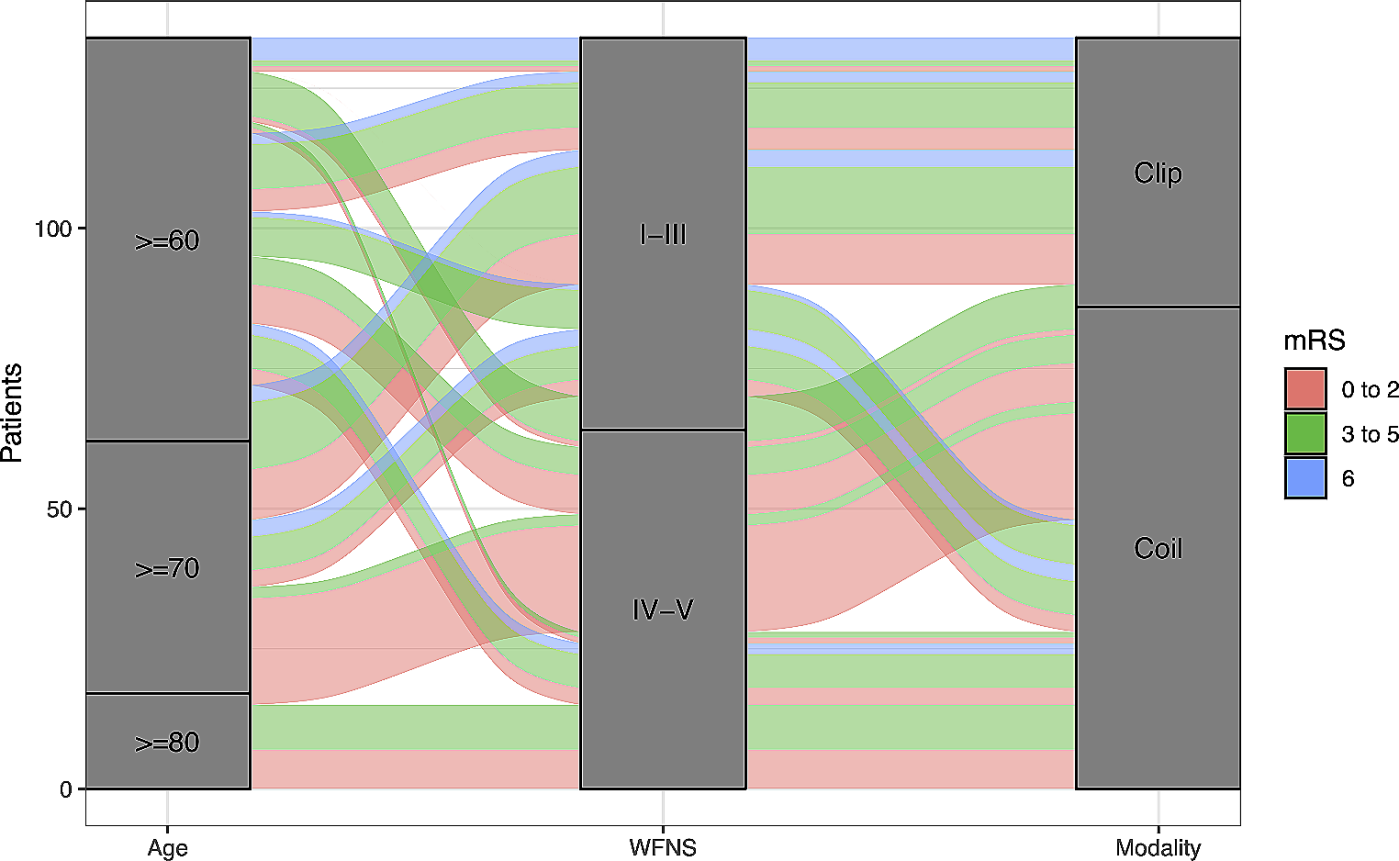
Alluvial plot to visualize the journey amongst the elderly patients with aneurysmal subarachnoid hemorrhage with the three mRS categories – 0 to 2, 3 to 5 and 6 – at 3 months (*N* = 134). Journeys of the various mRS categories are represented by a “track” that flows through the stages of independent predictors of good functional outcomes. The columns indicate whether or not the patients had those predictors. The thickness of the track corresponds to the number of respondents who shared the same mRS categories. mRS = modified Rankin scale. WFNS = World Federation of Neurosurgical Societies

On univariable analyses, age (*p* = 0.003), admission WFNS score I – III (*p* = 0.001), presence of acute hydrocephalus (*p* < 0.001) and coiling (*p* = 0.018), were found to be significant predictors of good functional outcomes mRS scores 0–2, at 3 months. On multivariable analysis, only age (*p* < 0.001), admission WFNS score I – III (*p* = 0.009), and coiling (*p* = 0.012) remained statistically significant predictors of good functional outcomes mRS scores 0–2, at 3 months. Figure 2 illustrates the journey amongst the elderly patients with aSAH in terms of their functional outcome by mRS scores.

On univariable analyses, age (*p* = 0.013), admission WFNS score I – III (*p* < 0.001), and presence of acute hydrocephalus (*p* = 0.005) were found to be significant predictors of good functional outcomes mRS scores 0–2, at 6 months. On multivariable analysis, only age (*p* = 0.005) and admission WFNS score I – III (*p* = 0.005) remained statistically significant predictors of good functional outcomes mRS scores 0–2, at 6 months.

On univariable analyses, admission WFNS score (*p* = 0.004), aneurysm size ≥7 mm (*p* = 0.043), treatment modality (*p* = 0.026), large infarct requiring decompressive craniectomy (*p* = 0.026), and delayed hydrocephalus requiring ventriculoperitoneal shunt (*p* = 0.018) were found to be significant predictors of in-hospital mortality. On multivariable analysis, admission WFNS score (*p* = 0.008), treatment modality (*p* = 0.041), and delayed hydrocephalus requiring ventriculoperitoneal shunt (*p* = 0.018) remained statistically significant predictors of in-hospital mortality. Sensitivity analyses demonstrated stable and robust risk estimates following application of multiple imputation to account for missing covariates and outcome data.

### Subgroup analysis

Within the WFNS score IV – V subgroup, the beneficial effect of coiling over neurosurgical clipping in terms of good mRS scores at 3-month (*p* = 0.090) and 6-month (*p* = 0.214) and in-hospital mortality (*p* = 0.098) diminished. Within the age 60–69 subgroup, the beneficial effect of coiling over neurosurgical clipping in terms of good mRS scores at 3-month was retained (*p* = 0.038), but lost for good mRS scores at 6-month (*p* = 0.175) and in-hospital mortality (*p* = 0.509).

## Discussion

Our findings demonstrate that endovascular coiling compared with neurosurgical clipping was associated with higher incidences of good functional outcomes and lower incidences of in-hospital mortality, with no significant differences in the incidence of complications, in terms of aneurysm rebleed, delayed hydrocephalus, delayed ischemic neurological deficit and venous thromboembolism. However, more patients in the clipping group developed large infarcts requiring decompressive craniectomy. A lower age, favorable admission WFNS scores and endovascular treatment were consistently identified as independent predictors of good functional outcomes in elderly patients with aSAH, whilst the latter two factors were also independent predictors of in-hospital mortality.

Our study avoided preselection by including patients who, because of their clinical status, did not receive treatment of the ruptured aneurysm. Numerous studies have reported reasonable outcomes for elderly patients with aSAH, but older and poor-grade patients are often underrepresented in these analyses [4, 10, 11, 14, 15], as they are shown to be predictive of unfavorable outcomes and mortality [5, 12, 15, 24]. An important consideration in the controversial debate around treatment of elderly patients with poor-grade aSAH is the possibility of increasing the number of dependent patients [1–3]. The elderly are a heterogenous population with the hexegenarians differing clinically from the octogenarian subgroup. A consensus for an age cap for maximal aneurysm treatment remains to be achieved for aSAH. The clinical implications of this are that maximal active aneurysm treatment should be recommended to the young (aged 60–79) elderly subgroup, especially if they are alert at the time of presentation, given the likelihood of positive short term outcomes at 3 months. Indeed this is supported by Goldberg et al. who showed that despite its high initial mortality, maximal treatment of aSAH in the elderly resulted in a reasonable proportion of favorable outcomes [12]. Such a trend is also reflective of improvements in coiling technology and technical know-how which underpin the improvements in patient outcomes. This is further reinforced by an observed increase in the percentage of aSAH cases treated with coiling at our institution from 42% in 2009, to 63% in 2019, reflecting a gradual shift in treatment paradigm in favor of coiling [25]. Notwithstanding, the better patient outcomes after coiling compared with clipping could also be partly explained by the selection bias our included patients were inevitably subjected to [26]. At our institution, all aSAH patients are first considered for coiling, and only proceeds to clipping if not amendable to endovascular means. Hence, the general pool of clipped aneurysms were more complex with poorer grades at admission to begin with, predisposing to postoperative complications [17, 27]. However, these were accounted for in our regression and subgroup analyses.

However, we should note the possibility of coiling losing its advantage over conventional clipping in the long term. Our results showed that elderly aSAH patients who were treated endovascularly had superior functional outcomes compared to those treated neurosurgically in the short term at 3 months post discharge, but there was no statistical significance in outcomes between these two groups by 6 months post discharge. This could indicate the possibility of coiling and clipping yielding comparable outcomes in the long-term, beyond our 6-month follow-up period. Long-term follow-up of the ISAT trial lends some support to this view, reporting a comparable rate of dependency in the coiling and clipping groups in the long run [17, 27]. Moving forward, there is a need for larger prospective trials to shed light on current evidence on this important clinical topic [7]. With increasing evidence supporting the safety and effectiveness of maximal treatment in elderly patients with aSAH [4, 10, 12, 27–29], there may finally be sufficient clinical equipoise to warrant a randomized prospective trial that could help to address the question at hand [30].

### Limitations

Our findings contribute to addressing this gap in literature on the appropriate management of elderly patients with aSAH, as long as they are interpreted judiciously with the following limitations in mind. The limitations of our study stem from its retrospective nature. First, being a retrospective review, non-standardized documentation of medical records could have resulted in bias in the collected data. This limitation was mitigated by the relatively small proportion of patients with incomplete documentation or lost to follow-up, minimising attrition bias. In addition, application of multiple imputation preserving sample size and statistical power, demonstrated stable and robust risk estimates [21–23]. Secondly, the moderate sample size in our cohort limited further subgroup analyses to delineate the benefits of coiling over neurosurgical clipping. Despite additional analyses, our study may have been biased by residual confounders including baseline characteristics such as excessive alcohol consumption or sarcopenia which have been shown to be associated with poorer outcomes, especially in the elderly [31, 32]. For example, Katsuki et al. showed that temporal muscle thickness and area, as indicators of sarcopenia, would indicate premorbid mRS. Further work from us could include sarcopenia as potentially useful to decide surgical indication and to predict outcome after aneurysm treatment in the elderly [32]. Future work could also investigate for particular complications such as seizures and pneumonia. Ryttlefors demonstrated that, in the subgroup of elderly aSAH patients treated in the ISAT, frequency of epilepsy and pneumonia was greater after neurosurgical clipping than after coiling. They had attributed the greater rates of epilepsy to craniotomy, aneurysm dissection, and the use of self-retaining brain retractors and greater rates of pneumonia to prolonged artificial ventilation, and prolonged bed rest in patients who had undergone neurosurgical clipping [27]. Finally, our study was conducted using data from three tertiary institutions, with several different surgeons attending to the patients. There may have been slight differences in management despite a largely standardized protocol at our institutions. However, this is reflective of real-world practice and hence enhances the applicability of our findings to the general cohort of elderly patients with aSAH. The next phase of the study would be to follow-up to at least one year which would be especially valuable, in investigating the rate of complete aneurysmal occlusion and need for reoperation.

## Conclusion

Endovascular coiling, compared with neurosurgical clipping, is associated with significantly better short term outcomes in carefully selected elderly patients with aSAH but its benefits diminishes at 6 months. Maximal intervention is recommended for aSAH in the young elderly age group and those with favorable WFNS scores.

## Data Availability

No datasets were generated or analysed during the current study.

## References

[1] Kontis V et al (2017) Future life expectancy in 35 industrialised countries: projections with a bayesian model ensemble. Lancet 389(10076):1323–1335 28236464 10.1016/S0140-6736(16)32381-9 PMC5387671

[2] de Rooij NK et al (2007) Incidence of subarachnoid haemorrhage: a systematic review with emphasis on region, age, gender and time trends. J Neurol Neurosurg Psychiatry 78(12):1365–1372 17470467 10.1136/jnnp.2007.117655 PMC2095631

[3] Lee KS et al (2021) Radiological surveillance of small unruptured intracranial aneurysms: a systematic review, meta-analysis, and meta-regression of 8428 aneurysms. Neurosurg Rev 44(4):2013–2023 33094423 10.1007/s10143-020-01420-1

[4] Ryttlefors M et al (2010) Neurointensive care is justified in elderly patients with severe subarachnoid hemorrhage–an outcome and secondary insults study. Acta Neurochir (Wien), 152(2): p. 241-9; discussion 249 10.1007/s00701-009-0496-x 19707714

[5] Proust F et al (2010) Interdisciplinary treatment of ruptured cerebral aneurysms in elderly patients. J Neurosurg 112(6):1200–1207 19961311 10.3171/2009.10.JNS08754

[6] Lee KS et al (2024) Effectiveness of cerebrospinal fluid lumbar drainage among patients with Aneurysmal Subarachnoid Hemorrhage: an updated systematic review and Meta-analysis. World Neurosurg 10.1016/j.wneu.2024.01.062 38246528

[7] Lee KS et al (2023) Antiplatelet therapy in aneurysmal subarachnoid hemorrhage: an updated meta-analysis. Neurosurg Rev 46(1):221 37665377 10.1007/s10143-023-02120-2 PMC10477151

[8] Hoh BL et al (2023) Guideline for the Management of Patients With Aneurysmal Subarachnoid Hemorrhage: A Guideline From the American Heart Association/American Stroke Association. Stroke 10.1161/STR.0000000000000436 37212182

[9] Johansson M et al (2001) Changes in intervention and outcome in elderly patients with subarachnoid hemorrhage. Stroke 32(12):2845–2949 11739985 10.1161/hs1201.099416

[10] Lanzino G et al (1996) Age and outcome after aneurysmal subarachnoid hemorrhage: why do older patients fare worse? J Neurosurg 85(3):410–418 8751625 10.3171/jns.1996.85.3.0410

[11] Nieuwkamp DJ et al (2006) Subarachnoid haemorrhage in patients > or = 75 years: clinical course, treatment and outcome. J Neurol Neurosurg Psychiatry 77(8):933–937 16638789 10.1136/jnnp.2005.084350 PMC2077608

[12] Goldberg J et al (2018) Survival and Outcome after Poor-Grade Aneurysmal Subarachnoid Hemorrhage in Elderly patients. Stroke 49(12):2883–2889 30571422 10.1161/STROKEAHA.118.022869

[13] Pavelka M et al (2023) Vasospasm risk following aneurysmal subarachnoid hemorrhage in older adults. J Neurosurg, : p. 1–9 10.3171/2023.3.JNS222720 PMC11122688 37119113

[14] Suzuki Y et al (2016) Results of clipping surgery for Aneurysmal Subarachnoid Hemorrhage in Elderly patients aged 90 or older. Acta Neurochir Suppl 123:13–16 27637623 10.1007/978-3-319-29887-0_2

[15] Hironaka K et al (2020) Outcomes in Elderly Japanese patients treated for Aneurysmal Subarachnoid Hemorrhage: a Retrospective Nationwide Study. J Stroke Cerebrovasc Dis 29(6):104795 32222416 10.1016/j.jstrokecerebrovasdis.2020.104795

[16] Molyneux AJ et al (2005) International subarachnoid aneurysm trial (ISAT) of neurosurgical clipping versus endovascular coiling in 2143 patients with ruptured intracranial aneurysms: a randomised comparison of effects on survival, dependency, seizures, rebleeding, subgroups, and aneurysm occlusion. Lancet 366(9488):809–817 16139655 10.1016/S0140-6736(05)67214-5

[17] Molyneux AJ et al (2015) The durability of endovascular coiling versus neurosurgical clipping of ruptured cerebral aneurysms: 18 year follow-up of the UK cohort of the International Subarachnoid Aneurysm Trial (ISAT). Lancet 385(9969):691–697 25465111 10.1016/S0140-6736(14)60975-2 PMC4356153

[18] Spetzler RF et al (2015) The Barrow ruptured Aneurysm Trial: 6-year results. J Neurosurg 123(3):609–617 26115467 10.3171/2014.9.JNS141749

[19] Wiebers DO et al (2003) Unruptured intracranial aneurysms: natural history, clinical outcome, and risks of surgical and endovascular treatment. Lancet 362(9378):103–110 12867109 10.1016/s0140-6736(03)13860-3

[20] Organization WH *Ageing*. 13 June 2023]; Available from: https://www.who.int/health-topics/ageing#tab=tab_1

[21] Saffari SE et al (2022) Proper use of multiple imputation and dealing with missing Covariate Data. World Neurosurg 161:284–290 35505546 10.1016/j.wneu.2021.10.090

[22] Rubin DB, Schenker N (1991) Multiple imputation in health-care databases: an overview and some applications. Stat Med 10(4):585–598 2057657 10.1002/sim.4780100410

[23] White IR, Royston P, Wood AM (2011) Multiple imputation using chained equations: issues and guidance for practice. Stat Med 30(4):377–399 21225900 10.1002/sim.4067

[24] Park J et al (2014) Critical age affecting 1-year functional outcome in elderly patients aged ≥ 70 years with aneurysmal subarachnoid hemorrhage. Acta Neurochir (Wien) 156(9):1655–1661 24950994 10.1007/s00701-014-2133-6

[25] Koh KM et al (2013) Management of ruptured intracranial aneurysms in the post-ISAT era: outcome of surgical clipping versus endovascular coiling in a Singapore tertiary institution. Singap Med J 54(6):332–338 10.11622/smedj.2013127 23820544

[26] Lee KS et al (2022) The evolution of intracranial aneurysm treatment techniques and future directions. Neurosurg Rev 45(1):1–25 33891216 10.1007/s10143-021-01543-z PMC8827391

[27] Ryttlefors M et al (2008) International subarachnoid aneurysm trial of neurosurgical clipping versus endovascular coiling: subgroup analysis of 278 elderly patients. Stroke 39(10):2720–2726 18669898 10.1161/STROKEAHA.107.506030

[28] Braun V et al (2005) Treatment and outcome of aneurysmal subarachnoid haemorrhage in the elderly patient. Neuroradiology 47(3):215–221 15912417 10.1007/s00234-005-1356-x

[29] Koffijberg H, Buskens E, Rinkel GJ (2011) Aneurysm occlusion in elderly patients with aneurysmal subarachnoid haemorrhage: a cost-utility analysis. J Neurol Neurosurg Psychiatry 82(7):718–727 19965848 10.1136/jnnp.2009.185660

[30] Zumofen DW et al (2018) Factors associated with clinical and radiological status on admission in patients with aneurysmal subarachnoid hemorrhage. Neurosurg Rev 41(4):1059–1069 29428981 10.1007/s10143-018-0952-2

[31] Juvela S (1992) Alcohol consumption as a risk factor for poor outcome after aneurysmal subarachnoid haemorrhage. BMJ 304(6843):1663–1667 1633519 10.1136/bmj.304.6843.1663 PMC1882384

[32] Katsuki M et al (2019) Clinical characteristics of aneurysmal subarachnoid hemorrhage in the elderly over 75; would temporal muscle be a potential prognostic factor as an indicator of Sarcopenia? Clin Neurol Neurosurg 186:105535 31569058 10.1016/j.clineuro.2019.105535

